# Vitamin D supplementation: less controversy, more guidance needed

**DOI:** 10.12688/f1000research.8863.1

**Published:** 2016-08-17

**Authors:** Caroline S. Stokes, Frank Lammert

**Affiliations:** 1 Department of Medicine II, Saarland University Medical Center, Saarland University, Homburg, Germany

**Keywords:** vitamin D supplementation, dosing regimens, calcium absorption

## Abstract

Vitamin D is a secosteroid hormone with multiple functions that extend beyond the regulation of intestinal calcium absorption. In recent years, the publication of research articles investigating associations between vitamin D status and health has reached an all-time high, and an increase in supplementation studies has followed. Given the pleiotropic effects of vitamin D, the scientific focus has gone beyond its known classic benefits on skeletal health to include diabetes and cardiovascular, neurological, respiratory, renal, and liver diseases, yet numerous conflicting findings continue to emerge. This review presents some examples of recent work within the context of controversies surrounding vitamin D and highlights key factors that should be considered when designing vitamin D supplementation regimens.

## Introduction

Vitamin D metabolites encompass a group of secosteroid compounds whose primary function is intestinal calcium absorption regulation. Though classified as a vitamin, contribution from food consumption (that is, mainly vitamin D _2_) is relatively limited in some geographical locations unless food has been fortified, such as in some industrialised countries of the northern hemisphere. Vitamin D _3_ is also photo-produced in skin following ultraviolet B exposure ^1^. In order for vitamin D to be used by the body, it is transported to the liver (bound mainly to Gc-globulin) ^2^ and undergoes 25-hydroxylation, yielding the 25-hydroxyvitamin D metabolite, the concentrations of which can be readily measured in blood. This metabolite then undergoes renal hydroxylation, creating the most biologically active vitamin D metabolite, 1,25-dihydroxyvitamin D, which applies its functions through the gatekeeper to multiple cells in the body, the nuclear vitamin D receptor.

There are various prevalence ranges reported in the literature for vitamin D deficiency, which are partially attributed to the different definitions of what constitutes a vitamin D deficiency (for example, serum 25-hydroxyvitamin D concentrations of less than 20 ng/ml or of less than 12 ng/ml); thus, research in this area is fraught with conflicting findings ^3, 4^. Existing controversy has led to a degree of confusion surrounding the significance of vitamin D in health and disease as well as uncertainty in deciding which replacement strategies are the most efficacious, and this hampers clinical decision-making. Furthermore, optimal vitamin D concentrations have yet to be agreed on, and accurate and precise methodologies to quantify vitamin D metabolites are still being developed. Though skeletal benefits of vitamin D supplementation have been documented, studies failing to observe such benefits have also been published ^5^. In fact, evidence of harm has even been reported with high-dose vitamin D supplementation within the context of falls risk in the elderly ^6^. The same holds true for other pleiotropic (for example, non-skeletal) benefits of vitamin D, such as diabetes and cardiovascular, respiratory, neurological, renal, and liver diseases, and studies report both positive effects as well as lack of effects ^7– 11^. An evaluation of the efficacy of vitamin D supplementation in various diseases is not the focus of this review, as such appraisals have been extensively carried out ^12– 14^. Moreover, within the same studies, patients receiving the identical vitamin D dosing regimen may respond very differently to supplementation and some do not display increases in serum vitamin D, thus influencing the primary endpoint being investigated. This leads to the following questions: how should we supplement with vitamin D in a responsible and efficacious manner, and who is best suited to receive such supplementation? These areas of controversy are discussed below.

## Determining vitamin D status and defining optimal concentrations

Vitamin D status is usually determined by using concentrations of serum 25-hydroxyvitamin D because this is the metabolite with the longest half-life and is directly linked to vitamin D _3_ substrate availability. The 25-hydroxyvitamin D metabolite can be quantified with a variety of methods ranging from immunoassays and chemiluminescence assays to liquid chromatography-mass spectrometry techniques ^15^. These different methods, however, vary in their estimation of serum vitamin D concentrations: 10% to 20% variability in vitamin D assays is detected even when the same samples are retested ^16^.

Liquid chromatography is currently considered the gold-standard method; however, assays such as immunoassays have shown comparable precision ^17^. One significant advantage with liquid chromatography techniques is that they enable the separation of vitamin D species and metabolites that cause interferences. One such example is the C-3 epimer of 25-hydroxyvitamin D, the functions of which remain unknown ^18^. This epimer is present in higher concentrations in infants and children as compared with adults yet, if not properly accounted for, might be included in 25-hydroxyvitamin D concentrations, and this could lead to overestimation of vitamin D levels ^18– 20^. Thus, even small changes in vitamin D concentrations, such as those that result from vitamin D supplementation, might not be adequately accounted for, given the variability in quantification from the various methods.

Currently, optimal serum vitamin D concentrations are controversial, and there is a discrepancy between two key guidelines. The Institute of Medicine recommends 20 ng/ml (50 nmol/l; 1 ng/ml = 0.40 nmol/l) as adequate on the basis of bone health studies ^16^, which is in contrast to the 30 ng/ml (75 nmol/l) urged by the US Endocrine Society ^21^. To achieve levels of the latter, approximately 4,000 IU of vitamin D would be required daily ^22^. However, the safety of such a dose needs to be confirmed in further controlled trials, as, for example, a higher risk of upper respiratory tract infections has been reported with 4,000 IU of vitamin D per day in asthma patients achieving circulating 25-hydroxyvitamin D levels of more than 30 ng/ml ^23^. It has even been suggested that there might be no “one fits all” cutoff that should be advocated, but rather disease-specific vitamin D levels ^24^. Currently, the most convincing evidence for vitamin D cutoffs exists for fracture risk reduction ^25, 26^. There is, for instance, evidence from a meta-analysis with well-conducted randomised controlled trials (RCTs) that optimal serum concentrations of 25-hydroxyvitamin D of between 24 ng/ml (60 nmol/l) and 38 ng/ml (95 nmol/l) are required to reduce the risk of falls by 19% ^27^. There is also evidence for additional health benefits of vitamin D on mortality ^28^ and upper respiratory tract infections ^29^; however, more studies are needed before disease-specific levels can be recommended and thus a more precise treatment approach to vitamin D deficiency implemented.

## Vitamin D dosing regimens

Once a therapeutic target has been decided on, the next step is to choose a dosing regimen. This depends on several factors, such as the type of vitamin D metabolite to administer, dose efficacy, severity of vitamin D deficiency, convenience for the dosing recipients, and safety. The two main contenders for vitamin D supplementation are vitamin D _2_ or vitamin D _3_. Further potent downstream metabolites such as calcifediol (25-hydroxyivtamin D) and calcitriol (1,25-dihydroxyvitamin D) are associated with the adverse events of hypercalciuria and hypercalcaemia and have to be monitored carefully ^6^. Vitamin D _3_ has shown superiority in terms of bioavailability and in consequently raising serum 25-hydroxyvitamin D concentrations, as shown in a meta-analysis of seven RCTs ^30^.

When to initiate supplementation is also a key consideration; for example, should patients undergoing certain invasive therapies receive vitamin D pre- or post-procedure? One recent example from the bariatric surgery field illustrates how supplementation with vitamin D before surgery leads to a greater likelihood of maintaining optimal serum 25-hydroxyvitamin D concentrations, which are often severely deficient in these patients because of the malabsorptive techniques used and poor absorption of fat-soluble nutrients ^31^. Generally, obese patients frequently display lower vitamin D concentrations, attributed to adipose tissue sequestering vitamin D ^32^. The American Society for Metabolic and Bariatric Surgery 2013 Guidelines ^33^ advocate raising blood 25-hydroxyvitamin D concentrations to above 30 ng/ml by administering a standard daily dose of 3,000 IU of vitamin D and using serial measurements to titrate the dose to optimise serum concentrations. However, these recommendations are not based on clinical outcome parameters, and it must be kept in mind that a 25-hydroxyvitamin D level of 30 ng/ml has yet to be fully substantiated as a well-established lower target value, yet it illustrates an important point: obese patients might generally benefit from higher supplementation doses ^34^ as compared with normal-weight patients because of extra adipose tissue (that is, with supplementation regimens guided by body weight) ^35^. In fact, Zittermann *et al.* ^35^ have established a formula for calculating vitamin D dosing regimens which takes into account body weight as well as age, initial circulating 25-hydroxyvitamin D concentrations, and type of vitamin D supplement (D _2_ or D _3_). Various nutrition societies (for example, in the US and in Europe) already recommend 600 to 800 IU of vitamin D daily to prevent vitamin D deficiency ^36, 37^. If higher doses should be recommended in the clinical setting, the efficacy and safety of these doses have to be proven by RCTs.

Finally, daily versus bolus (for example, monthly) feeding should also be considered. There is evidence that regular intake may be more beneficial, as this delivers a daily dose of vitamin D and thus can provide stable concentrations of circulating vitamin D, which appears to be particularly important for endocrine and autocrine functions ^38, 39^. There is also evidence that daily rather than bolus supplementation with vitamin D is more effective in preventing respiratory infection ^29^.

## Factors influencing response to supplementation

**Figure 1.**
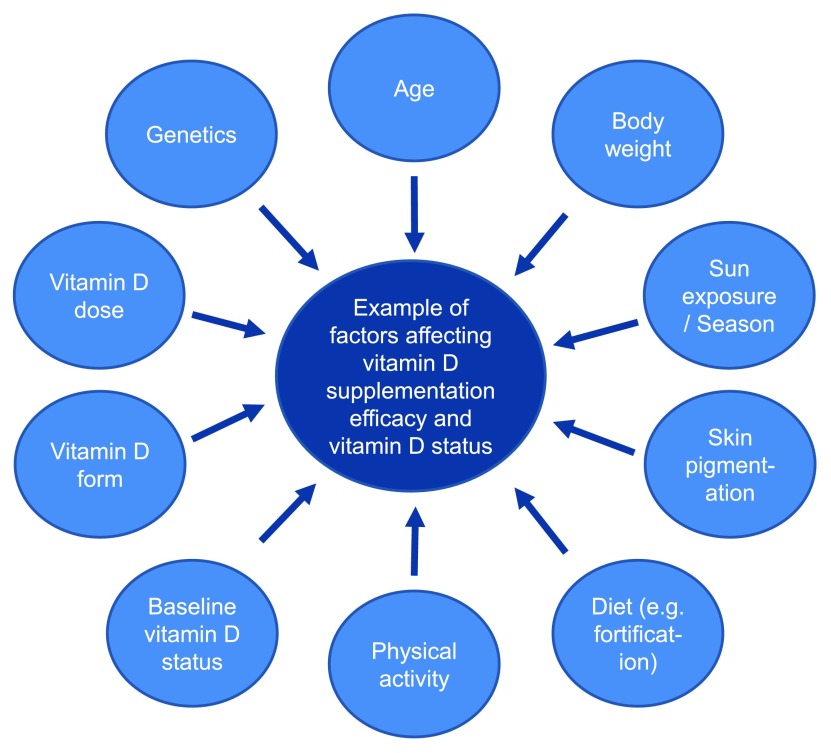
Factors that can influence vitamin D supplementation efficacy and vitamin D status.

Body weight, however, is not the only factor affecting the response to supplementation, as marked variability is often observed between patients during identical dosing regimens ^40^. Serum 25-hydroxyvitamin D concentrations are also affected by physical activity, skin pigmentation, age, and genetic factors, to name but a few factors ( Figure 1) ^1, 41, 42^. Wang *et al.* ^41^ identified three key vitamin D polymorphisms—group-specific component ( *GC*), 7-dehydrocholesterol reductase ( *DHCR7*), and cytochrome P450, family 2, subfamily R polypeptide 1 ( *CYP2R1*)—that were involved in vitamin D synthesis, hydroxylation, and transport and thus were shown to influence circulating 25-hydroxyvitamin D concentrations. Moreover, these polymorphisms have been shown to influence response to vitamin D supplementation ^43^. The *GC* gene encodes the vitamin D-binding protein Gc-globulin. In 2013, Powe *et al.* measured lower blood concentrations of not only total 25-hydroxyvitamin D but also Gc-globulin in black as compared with white Americans and reported that bioavailable 25-hydroxyvitamin D did, in fact, not differ between races in the study ^44^. They speculated that this was likely due to the lower levels of circulating Gc-globulin in the black Americans, thus suggesting that using total 25-hydroxyvitamin D as a marker for vitamin D status might be misleading. However, this conclusion has recently been questioned, with differences in assays used (for example, monoclonal versus polyclonal) being critical ^45^.

When response to supplementation is assessed, it is most important to consider the season because time of year could confound the relationship, as could study participants going abroad to sunny locations. Indeed, sun exposure will almost always confound findings assessing serum response to vitamin D supplementation because it is the primary source of vitamin D and it is difficult to accurately account for, although progress in the development of devices such as wrist actigraphy that can capture light exposure as well as physical activity might make this easier to document ^1, 46^. This can be statistically controlled for to a certain extent in research settings, but it should also be considered in clinical practice. Likewise, vitamin D food fortification practices might need to be taken into account (even though contribution to vitamin D status from such foods is much smaller). Nevertheless, the extent of such contributions will be country specific since in Germany, for example, there is very scant food fortification in comparison with the United States ^47, 48^. Such factors are relevant because very high serum 25-hydroxyvitamin D concentrations have been linked to adverse events in some studies ^49^; however, this has not been shown in other trials and in several meta-analyses, for example ^50, 51^.

Indeed, a recent RCT in *JAMA Internal Medicine* reported an increased risk of falls with high-dose vitamin D supplementation in patients over the age of 70 years ^6^. At baseline, 200 patients had serum 25-hydroxyvitamin D concentrations of below 20 ng/ml and were given one of three dosing regimens at monthly intervals: 60,000 IU of vitamin D _3_ (equates to 2,000 IU/day) or 24,000 IU of vitamin D _3_ (equates to 800 IU/day) either alone or combined with 300 μg of calcifediol. Both the vitamin D _3_ at 60,000 IU and the 24,000 IU plus 300 μg of calcifediol regimens displayed higher efficacy in increasing serum 25-hydroxyvitamin D concentrations to the 30 ng/ml range in comparison with the group receiving 24,000 IU of vitamin D _3_ only. Both of these regimens, however, were associated with a significantly ( *P* = 0.048) greater risk of falls over a follow-up period of 12 months: 66.9% (95% confidence interval [CI] 54.4%–77.5%) and 66.1% (95% CI 53.5%–76.8%), respectively, as compared with the group that received 24,000 IU only (47.9%; 95% CI 35.8%–60.3%). In this study, only patients with inadequate serum 25-hydroxyvitamin D concentrations (and not patients with values considered to be in the normal range) received supplementation ^52^.

Knowledge of a patient’s baseline vitamin D level is of utmost importance but is not always taken into account in studies ^53, 54^. In fact, this has been suggested as a causative factor for many negative studies since patients with normal or near-normal levels receiving vitamin D supplementation are unlikely to exhibit physiological benefits ^55^. This notion was summarised by Heaney in an editorial ^53^, in which he emphasises the “dose-response to supplementation curve”, stating that nutrients such as vitamin D do not behave like drugs and that one needs to start with depleted stores to observe benefits from supplementation.

Heaney also reiterates that the dosage needs to be sufficient to enable the measured vitamin D concentrations to increase enough during supplementation so as to push patients into replenishment levels. A minor increase in serum concentrations is unlikely to result in significant improvements. Such a finding was succinctly illustrated in the recent VITdAL-ICU RCT, in which 475 critically ill patients in the ICU were given a one-off high dose of 540,000 IU of vitamin D _3_ (either orally or via a nasogastric feeding tube), followed by a maintenance dose of 90,000 IU once a month for 5 months ^56^. Neither length of hospital stay nor 6-month mortality was reduced; however, significantly lower mortality was observed for patients with very low vitamin D baseline concentrations (that is, less than 10 ng/ml as compared with the placebo group [28.6% versus 46.1%; *P* = 0.04], with a hazard ratio of 0.56 [95% CI 0.35–0.90]). This study reported no serious adverse events, although 11% of patients receiving vitamin D _3_ displayed elevated serum calcium concentrations as compared with 2% in the placebo group.

Mortality has been linked to low vitamin D levels in previous studies, summarised in the recent meta-analyses ^57, 58^. Moreover, a Cochrane systematic review and meta-analysis reported the efficacy of vitamin D _3_ supplementation in reducing mortality in the elderly ^28^. As seen in the VITdAL trial, certain patients (for example, those with lower baseline vitamin D concentrations) may benefit more from supplementation. Indeed, lower serum 25-hydroxvitamin D concentrations have previously been linked to increased mortality in a meta-analysis of community-based cohort studies ^50^. A recent hypothesis based on data from the ESTHER (Epidemiological Study on Chances for Prevention, Early Detection, and Optimized Therapy of Chronic Diseases at Old Age) cohort study and CHANCES (Consortium on Health and Ageing: Network of Cohorts in Europe and the United States) suggests that adverse effects of vitamin D deficiency appear to apply mainly to diseased patients rather than to healthy populations (that is, at-risk persons), illustrating that vitamin D presents as a “resilience factor” in potentially fatal diseases ^59^. Therefore, it might be more appropriate to focus supplementation on such patients as they stand to receive the most health benefits. The controversy, however, continues as a reverse J-shaped curve has been reported recently in the Copenhagen vitamin D study (CopD study) with regard to serum 25-hydroxyvitamin D concentrations and mortality rates from cardiovascular disease ^49^. In this observational study, values at the lower extreme of approximately 5 ng/ml 25-hydroxyvitamin D levels for men and at the higher extreme of approximately 50 ng/ml for both men and women were linked to elevated risk of death. The concentrations that were deemed most optimal for reduced risk of death were centered around 28 ng/ml.

## Conclusions

The vitamin D field clearly has ongoing controversies that still need to be resolved in order to more effectively guide research and clinical practice with regard to vitamin D supplementation. Key areas include the refinement of the gold-standard methods for the quantification of vitamin D metabolites, ideally with widespread use of similar methods and assay standardization so as to make serum values directly comparable between studies and clinical centers. A consensus on acceptable cutoffs and on what constitutes adequate vitamin D levels is also needed, possibly avoiding a one-value-fits-all approach but rather tailoring it to the specific background conditions or disease states. In both clinical and research settings, it is crucial that patients have their vitamin D status checked prior to supplementation and at least once or, if costs permit, regularly throughout long-term supplementation (for example, every 6 months). Also imperative is that only those with depleted vitamin D stores receive supplementation so as to ensure treatment efficacy and to minimise the risk of adverse events, particularly when higher doses are administered.

Most importantly, interpretation of study endpoints needs to be conducted within the context of changes to serum 25-hydroxyvitamin D concentrations between baseline and post-supplementation rather than based on patient compliance. Factors that can affect vitamin D serum concentrations during supplementation, such as obesity, time of year, and likelihood of excessive sun exposure, also need to be considered. Daily dosing regimen ideally should be tailored to the individual or to the study population being included, and a more precise treatment strategy should be adopted and a vitamin D titration approach should be based on response of serum 25-hydroxyvitamin D concentrations.

## Abbreviations

CI, confidence interval; *GC*, group-specific component; RCT, randomised controlled trial; *DHCR7*, 7-dehydrocholesterol reductase; *CYP2R1*, cytochrome P450, family 2, subfamily R polypeptide 1.
